# More on the holographic Ricci dark energy model: smoothing Rips through interaction effects?

**DOI:** 10.1140/epjc/s10052-018-5773-5

**Published:** 2018-04-23

**Authors:** Mariam Bouhmadi-López, Ahmed Errahmani, Taoufik Ouali, Yaser Tavakoli

**Affiliations:** 1 Department of Theoretical Physics University of the Basque Country UPV/EHU, P.O. Box 644, 48080 Bilbao, Spain; 2 IKERBASQUE, Basque Foundation for Science, 48011 Bilbao, Spain; 3 Laboratory of Physics of Matter and Radiation, Mohammed First University, BP 717, Oujda, Morocco; 4 Faculty of Physics, University of Warsaw, Pasteura 5, 02-093 Warsaw, Poland; 5 School of Engineering Science, College of Engineering, University of Tehran, 11155-4563 Tehran, Iran; 6 School of Astronomy, Institute for Research in Fundamental Sciences (IPM), 19395-5531 Tehran, Iran

## Abstract

The background cosmological dynamics of the late Universe is analysed on the framework of a dark energy model described by an holographic Ricci dark energy component. Several kind of interactions between the dark energy and the dark matter components are considered herein. We solve the background cosmological dynamics for the different choices of interactions with the aim to analyse not only the current evolution of the universe but also its asymptotic behaviour and, in particular, possible future singularities removal. We show that in most of the cases, the Big Rip singularity, a finger print of this model in absence of an interaction between the dark sectors, is substituted by a de Sitter or a Minkowski state. Most importantly, we found two new * future* bouncing solutions leading to two possible asymptotic behaviours, we named Little Bang and Little Sibling of the Big Bang. At a Little Bang, as the size of the universe shrinks to zero in an infinite cosmic time, the Hubble rate and its cosmic time derivative blow up. In addition, at a Little sibling of the Big Bang, as the size of the universe shrinks to zero in an infinite cosmic time, the Hubble rate blows up but its cosmic time derivative is finite. These two abrupt events can happen as well in the past.

## Introduction

Several astrophysical observations (cf. for example supernovae type Ia [1, 2], cosmic microwave background [3], large scale structure [4], etc.) have confirmed that the universe is undergoing a state of accelerated expansion if homogeneity and isotropy are assumed on large scales. In addition, those experiments indicate that the matter content (i.e., the total mass-energy) of the universe, leading this accelerated expansion, must contain an exotic energy which is characterised with a sufficiently negative pressure. Dark energy (DE) is the most accepted hypothesis to explain the current observations, and constitutes roughly $70\%$ of the total matter content of the universe. However, so far there is no clear understanding of the true fundamental nature of DE. Indeed, the mysterious nature of dark energy is still among the long-standing problems in theoretical physics.

There are many dynamical models trying to explain the nature of DE [5, 6]. Among them, there is an attractive model which is inspired on the holographic principle rooted on quantum gravity [7–9]; it is the so called holographic dark energy [10, 11]. We next summarise the ideas behind this model. A well known fact is that the entropy of a given system with finite volume, $L^3$, has an upper bound which is not proportional to its volume, but rather to its surface area, $L^2$ [12, 13]. In addition, for an effective quantum field theory with a given ultra-violet (UV) cutoff, $M_{\text {UV}}$, the entropy of that system scales as $L^3M^3_{\text {UV}}$. Consequently, there is always a scale or a length where the quantum field theory with UV cutoff is expected to fail. This is expected to happen for large volumes or lengths. To solve this problem a link between UV and infrared (IR) cutoffs was proposed in [9]: $L^3M^4_{\text {UV}}\lesssim L M_{ \mathrm {P}}^2.$ By this mean the validity of the quantum field theory within this regime is assured. When the inequality is saturated, we can define an energy density which is inversely proportional to the square of the characteristic length of the system. These ideas have been applied to the universe giving rise to what is known as the holographic dark energy scenario [10]. The appealing holographic Ricci dark energy (HRDE) model consists in taking the square of the length characterising the Universe, *L*, as the inverse of the Ricci scalar curvature [14] (see also [15–21]).

It has been proven that the HRDE is suitable to describe the current acceleration of the universe as shown in [14, 22–25] . It has been equally shown that this model might induce a big rip (BR) singularity [14]; i.e., the scale factor, the Hubble parameter and its first cosmic time derivative reach very large values in a finite future cosmic time [26–28]. This model has been also constrained observationally [29]. More recent observational constraints on the HRDE can be found in Refs. [20, 21, 24, 30, 31] (cf. Ref. [32] for an extended list of references on the HRDE scenario).

On this paper, we intend to see how the BR^1^ present on the HRDE can be removed or appeased by the inclusion of interactions between cold dark matter (CDM) and the HRDE. An interaction on the dark sector and within the HRDE model has been previously analysed in [19–21, 30, 34–36] where the main goal of these papers was to study the adequacy (from an observational point of view) of these models to describe the late time acceleration rather than analysing the asymptotic behaviour of the universe. On this work, we will carry a thorough analytical analysis of the HRDE when a CDM and DE, given through the HRDE, are interacting. The goal of this work is to identify those interactions that are able to remove or smooth the BR. It is worth mentioning that an interaction on the dark sector has been favoured observationally [37, 38] and has been shown to be extremely helpful to mitigate the coincidence problem (cf. the recent review [39] on this topic).

The paper is organised as follows. In Sect. 2, we review the general setup of the HRDE model in a Friedmann-Lemaître-Robertson-Walker (FLRW) background in presence of an interaction term between DM and DE. In Sect.  3, we carry out a careful analysis of the asymptotic behaviour of the universe in this framework. Finally, in Sect. 4, we present our conclusions.

## General setup

We consider a spatially flat FLRW universe, filled with matter with the energy density $\rho$, whose evolution is described by the Friedmann equation:

2.1 $$\begin{aligned} 3M_{\text {P}}^{2}H^{2}\ =\ \rho , \end{aligned}$$

where $M_{\text {P}}$ is the reduced Planck mass. We assume that the total energy density, $\rho$, of the cosmic fluid is described through a CDM component with the energy density $\rho _{\mathrm {m}}$ and a HRDE component $\rho _{\mathrm {H}}$. The HRDE density is defined as [14]

2.2 $$\begin{aligned} \rho _{\mathrm {H}}\ =\ 3\beta M_{\text {P}}^{2}\left( \frac{1}{2}\frac{dH^{2}}{ dx}+2H^{2}\right) , \end{aligned}$$

where $x \equiv \ln (a/a_0)$ and $\beta$ is a positive dimensionless parameter that measures the strength of the holographic component. From now on a zero subindex stands for quantities evaluated at present.

It is convenient to rewrite the Friedmann equation (2.1) in terms of the dimensionless energy densities:

2.3 $$\begin{aligned} \Omega _{\mathrm {m}}&=\ \frac{\rho _{\mathrm {m}}}{3M_{\text {P} }^{2}H_{0}^{2}}, \nonumber \\ \Omega _{\mathrm {H}}&=\ \frac{\rho _{\mathrm {H}}}{3M_{\text {P} }^{2}H_{0}^{2}} =\beta \left( \dfrac{1}{2}\dfrac{dE^{2}}{dx}+2E^{2}\right) , \end{aligned}$$

where $E(z)=H/H_{0}$. Therefore, Eq. (2.1) becomes

2.4 $$\begin{aligned} E^{2}\ =\ \Omega _{\mathrm {m}}+\Omega _{\mathrm {H}}. \end{aligned}$$

This equation constrains the cosmological parameters of the model at present time, $x=0$, as

2.5 $$\begin{aligned} 1=\Omega _{\mathrm {m}_{0}}+\Omega _{\mathrm {H}_{0}}, \end{aligned}$$

in which the present value of the dimensionless Hubble rate *E* and its derivative with respect to *x* are governed by the following equations:

2.6 $$\begin{aligned} \left\{ \begin{array}{l} E(x=0)=1,\\ dE/dx|_{x=0}=-2+\frac{\Omega _{\mathrm {H}_{0}}}{\beta }\ . \end{array} \right. \end{aligned}$$

In addition, at present time, the deceleration parameter $q=-(1+\frac{1}{E} dE/dx)$ reads,

2.7 $$\begin{aligned} q_{0}=1-\frac{\Omega _{\mathrm {H}_{0}}}{\beta }\ , \end{aligned}$$

which must be negative (i.e., $q_{0}<0$) because the universe is accelerating currently. So that, the holographic parameter is constrained through the inequality

2.8 $$\begin{aligned} 0<\beta <\Omega _{\mathrm {H}_{0}}. \end{aligned}$$

A characteristic of the HRDE model is that the energy density of any matter component is self-conserved. However, we will further assume in this model an interaction between the energy densities of CDM and the HRDE components. Therefore, the corresponding conservation equations read

2.9 $$\begin{aligned}&\dot{\rho }_{\mathrm {H}} + 3H(1+\omega _{\mathrm {H}})\rho _{\mathrm {H}} =-Q\ , \end{aligned}$$

2.10 $$\begin{aligned}&\dot{\rho }_{\mathrm {m}} + 3H\rho _{\mathrm {m}} = Q, \end{aligned}$$

where the function *Q* denotes the interaction between the energy density of CDM and the holographic dark energy density components. Furthermore, positive *Q* represents energy transfer from CDM to DE, and vice versa for negative *Q*.

### General equations in the presence of interaction

It is expected physically and also from Eqs. (2.9) and (2.10) that the interaction is defined through the energy densities involved in the system, in particular *Q* should be a function of the energy densities of CDM and HRDE components multiplied by a quantity with the unit of inverse of time. For convenience, we choose the Hubble rate *H* as the characteristic magnitude with units of inverse of time, and hence ${Q=Q(H\rho _{\mathrm {m} },H\rho _{\mathrm {H}}, H\rho _{\mathrm {c}})}$ where $\rho _{\mathrm {c}}=3M_P^2H_0^2$ is the critical energy density. Since the value of the interaction parameter *Q* is small (cf. Ref. [39]), a power law expansion of *Q* in terms of the energy densities of the system is doable. The first order terms of this interaction; i.e. linear interaction, corresponds to

2.11 $$\begin{aligned} Q\ \simeq \ \lambda _{\mathrm {m}}H\rho _{\mathrm {m}}+\lambda _{\mathrm {H}}H\rho _{ \mathrm {H}}+{\lambda _{\mathrm {c}}H\rho _{\mathrm {c}}}\ , \end{aligned}$$

where $\lambda _{\mathrm {m}}$, $\lambda _{\mathrm {H}}$ and $\lambda _{\mathrm {c}}$ are constants.

Substituting Eq. (2.11) in Eq. (2.10), and replacing the energy densities in terms of dimensionless parameters, the conservation equation ( 2.10) can be written as

2.12 $$\begin{aligned} \dfrac{d\Omega _{\mathrm {m}}}{dx}=-\Omega _{\mathrm {m}}\left( 3-\lambda _{ \mathrm {m}}+\lambda _{\mathrm {H}}\right) +\lambda _{\mathrm {H}}E^{2}+{\lambda _{\mathrm {c}}}. \end{aligned}$$

On the other hand, the Friedmann equation (2.4) can be rewritten as a differential equation of the dimensionless Hubble rate *E* with respect to *x* as

2.13 $$\begin{aligned} \dfrac{dE^{2}}{dx}=2\left( \frac{1}{\beta }-2\right) E^{2}-\frac{2}{\beta } \Omega _{\mathrm {m}}. \end{aligned}$$

Notice that the equations (2.12) and (2.13) form a system of coupled equations. However, by differentiating both sides of Eq. (2.13) with respect to *x*, and using Eq. ( 2.12), we obtain a second order differential equation for $E^2$:

2.14 $$\begin{aligned} \frac{d^{2}E^{2}}{dx^{2}}&= -{ 2\frac{\lambda _{\mathrm {c}}}{\beta }} + 2\left[ \frac{3}{\beta }- 6 + \left( 2-\frac{1}{\beta }\right) \lambda _{ \mathrm {m}} - 2\lambda _{\mathrm {H}}\right] E^{2} \nonumber \\&\quad + \left( \frac{2}{\beta }+\lambda _{\mathrm {m} }-\lambda _{\mathrm {H}}-7\right) \frac{dE^{2}}{dx} ~\cdot \end{aligned}$$

The total conservation law for the system is given by $\dot{\rho } +3H(\rho +\omega _{\mathrm {H}}\rho _{\mathrm {H}})=0$ where the total energy density is given by $\rho =3M_{\text {P}}^2H_0^2E^2$. The equation of state (EoS) for the HRDE is

2.15 $$\begin{aligned} \omega _{\mathrm {H}}&=\ -\frac{1}{\Omega _{\mathrm {H}}}\left( E^2+\frac{1}{3} \frac{dE^2}{dx}\right) . \end{aligned}$$

This EoS can be rewritten by using Eqs. (2.3)-(2.4) as

2.16 $$\begin{aligned} \omega _{\mathrm {H}}&=\ \frac{1}{3}\left( \frac{\Omega _{\mathrm {m}}}{\Omega _{ \mathrm {H}}}+1-\frac{2}{\beta }\right) \nonumber \\&=\ \frac{1}{3}\left( \frac{E^{2}}{\Omega _{\mathrm {H}}}-\frac{2}{\beta } \right) . \end{aligned}$$

Notice that Eq. (2.16) is valid for any HRDE model where the total energy density of the universe is conserved.

In order to study the behaviour of the universe within the context of the HRDE, we will analyse the solutions of the differential equation (2.14) which depend on $\lambda _{\mathrm {m}}$, $\lambda _{\mathrm {H}}$, $\lambda _{\mathrm {c}}$ and $\beta$ parameters. We remind that we are interested in analysing the phase space of the parameters of the model for which the universe has a smooth future behaviour.

### Expected behaviour: general solutions

The general solution of the equation (2.14) can be written as:

2.17 $$\begin{aligned} E^{2}(x)\ =\ \mathbf {A}_{+}e^{\sigma _{+}x}+\mathbf {A}_{-}e^{\sigma _{-}x} + {\Lambda _{\mathrm {c}}}~, \end{aligned}$$

with $\mathbf {A}_{\pm }$ being constants,

2.18 $$\begin{aligned} {\Lambda _{\mathrm {c}}\ :=\ \frac{\lambda _{\mathrm {c}}}{3-6\beta +(2\beta -1)\lambda _{\mathrm {m}}-2\lambda _{\mathrm {H}}\beta }} ~, \end{aligned}$$

and

2.19 $$\begin{aligned}&\sigma _{\pm } := \sigma _{0}\pm \frac{\sqrt{\Delta }}{2\beta }, \nonumber \\&\mathrm {where} \quad \sigma _{0} = \frac{1}{2\beta }\big [2+(\lambda _{ \mathrm {m}}-\lambda _{\mathrm {H}}-7)\beta \big ]. \quad \quad \end{aligned}$$

The parameter $\Delta$ reads

2.20 $$\begin{aligned} \Delta = \left[ \Big (\lambda _{\mathrm {m}} + \lambda _{\mathrm {H}} + 1 -\frac{2 }{\beta }\Big )^2 - 4\lambda _{\mathrm {H}}(\lambda _{\mathrm {m}}+1)\right] \beta ^2. \end{aligned}$$

On the other hand, for the case in which $$\Delta =0$$ and $$\sigma _0\ne 0$$, the solution for $$E^{2} $$ reads

2.21 $$\begin{aligned} E^{2}(x)\ =\ (\mathbf {A}_{0}+\mathbf {A}_{1}x)e^{\sigma _{0}x}+{\Lambda _{\mathrm {c}}}~, \end{aligned}$$

where $\mathbf {A}_{0}$ and $\mathbf {A}_{1}$ are constants. The Hubble rate $E^{2}$ at the present time must satisfy the conditions of Eq. (2.6), i.e., the constants $\mathbf {A}_{\pm }$, $\mathbf {A}_{0}$ and $\mathbf {A}_{1}$ can be expressed in terms of $q_{0}$, Eq. (2.7), as

2.22 $$\begin{aligned}&\mathbf {A}_{\pm }= \pm \frac{2(1+q_{0})+{\sigma _{\mp }(1-\Lambda _{\mathrm {c}})}}{\sigma _{-}-\sigma _{+}}\ , \end{aligned}$$

2.23 $$\begin{aligned}&\mathbf {A}_{0}= 1-{\Lambda _{\mathrm {c}}}~, \end{aligned}$$

2.24 $$\begin{aligned}&\mathbf {A}_{1}= -2(1+q_{0})+\sigma _{0}({\Lambda _{\mathrm {c}}}-1) \ . \end{aligned}$$

Furthermore, by substituting the solutions (2.17) and (2.21) in the second equation of (2.3), the dimensionless HRDE densities read, respectively

2.25 $$\begin{aligned} \Omega _{\mathrm {H}}= & {} \ \frac{\beta \mathbf {A}_{+}}{2}\left( \sigma _{+}+4\right) e^{\sigma _{+}x} \nonumber \\&\quad + \frac{\beta \mathbf {A}_{-}}{2}\left( \sigma _{-}+4\right) e^{\sigma _{-}x}+{2\beta \Lambda _{\mathrm {c}}}~, \quad \quad \end{aligned}$$

and

2.26 $$\begin{aligned} { \Omega _{\mathrm {H}} = \frac{\beta }{2}\big [ (4+\sigma _0)(A_0+A_1x) + A_1 \big ] e^{\sigma _0x}+2\beta \Lambda _{\mathrm {c}}. } \quad \quad \end{aligned}$$

In the rest of this paper, we will analyse the solutions of Eqs. (2.17) and (2.21), and we will discuss their asymptotic behaviours, which depend on the choices of the holographic parameter $\beta$ and the interaction constants $\lambda _{\mathrm {m}}, \lambda _{ \mathrm {H}}$ and ${\lambda _{\mathrm {c}}}$. We will show that, in the far future, the fate of the universe may end up in one of the following states: a BR singularity [26–28]; a Minkowskian or a de Sitter behaviour; a little sibling of the BR (LSBR) [40]; we will as well show for the first time the presence of two other possible asymptotic behaviours corresponding to what we named the little bang (LB) and the little sibling of the big bang (LSBB) (cf. Sects. 3.2 and 3.3).

## Asymptotic behaviour and interaction effects

In this section, we will analyse the asymptotic behaviour of a FLRW filled with an HRDE fluid interacting with CDM within the model introduced on the previous section. In particular, we will analyse potential future singularities that might appear on the model by considering different interaction functions $Q\ne 0$. First of all, we will start with a brief review of the standard HRDE model in the absence of interactions.

### Standard HRDE model: $Q=0$

For vanishing parameters $\lambda _{\mathrm {m}}, \lambda _{\mathrm {H}}$ and $\lambda _{\mathrm {c}}$, in Eq. (2.11), there is no interaction (i.e., $Q=0$) and the standard HRDE model is recovered [14]. Then, by setting $\lambda _{\mathrm {m}}=\lambda _{\mathrm {H}}={\lambda _{\mathrm {c}}}=0$ in solution (2.17) we obtain the dimensionless Hubble rate *E*(*x*) (for the case $\beta \ne 2$) as (cf. Ref [14])

3.1 $$\begin{aligned} E^{2}(x)= & {} \ \frac{2q_{0}-1}{\beta -2}\beta \exp \left[ \left( \frac{2}{\beta }-4\right) x\right] \nonumber \\&\ \ \ -\frac{2(q_0-1)\beta +2}{\beta -2}\exp (-3x). \end{aligned}$$

The second term in Eq. (3.1) vanishes as $x\rightarrow +\infty$, and the Hubble rate is governed only by the first term. This indicates that, in the far future, the HRDE energy density mimicking matter (second term in Eq. (3.1)) is practically zero, and the universe converges asymptotically to a universe filled with the dominant HRDE component. Therefore, the properties of the solution (3.1) depend on the different ranges of the holographic parameter $\beta$. Let us summarise those behaviours as follows:

1. For $\beta >\frac{1}{2}$ ($\beta \ne 2$), the HRDE density tends to zero and the universe heads to a Minkowski state in the far future.
2. For $\beta =\frac{1}{2}$, the universe tends to a *de Sitter* state in the far future.
3. If $0<\beta <\frac{1}{2}$, the HRDE is dominant at late time. Using Eq. (2.16), the equation of state for HRDE energy can be written as
  3.2 $$\begin{aligned} \omega _{\mathrm {H}}=\frac{1}{3}\left( 1-\frac{2}{\beta }\right) , \end{aligned}$$
  which is always smaller than $-1$. Therefore, the HRDE component behaves as phantom-like matter at late time. By integrating Eq. (3.1), we obtain the evolution of the scale factor *a*(*t*):
  3.3 $$\begin{aligned}&a(t)=\Big [ CH_{0}(t-t_{0})+1\Big ] ^{\frac{\beta }{2\beta -1}}\ , \end{aligned}$$
  where
  3.4 $$\begin{aligned} \quad C:=\frac{2\beta -1}{\sqrt{\beta }}\sqrt{\frac{1-2q_{0}}{2-\beta }}. \end{aligned}$$
  Notice that, we have set $a(t_{0})=1$ as the value of the scale factor at present. On the other hand, from Eq. (2.25), we find that the dimensionless energy density of the HRDE increases negatively with time (since $\beta <\frac{1}{2}$). Then, at a finite time, namely $t_{\mathrm {BR}}$, the scale factor (Eq. (3.3)), the Hubble parameter and its cosmic time derivative blow up at $t_{\mathrm {BR}}$. Therefore, the universe hits a BR singularity at $t_{\mathrm {BR}}$. In fact, it can be seen that $t_{\mathrm {BR}}$ is a finite time and depends on the holographic parameter $\beta$.

Finally, for the case $\beta =2$ (which is not included in Eq. (3.1)), since $q_0<\frac{1}{2}$, the universe tends to a *Minkowski* state in the far future.

On the one hand, the latest observational data (e.g., Planck results [3]), implies that $\Omega _{m}\sim 0.308$ and $H_0=67.8~\mathrm{km}\cdot \mathrm{s}^{-1}\cdot \mathrm{Mpc}^{-1}$, thus, $q_0=-0.538$ (for a $\Lambda$ CDM universe). Then, by considering the condition (2.7), we can estimate the holographic parameter $\beta$ to be of the order $\beta \sim 0.448$. By setting $t_{0}=H_{0}^{-1}\sim 14.422$ Gyrs, we find that the BR would take place when $t_{\mathrm {BR}}=94.675$ Gyrs. On the other hand, we have seen that for $\beta \ge \frac{1}{2}$ there would be no abrupt events or singularities at late time. Given that we are interested in analysing DE singularities in this model, which are observationally favoured, from now on we will disregard values of $\beta$ such that $\beta \ge \frac{1}{2}$.

### The solution with $\Delta =\sigma _0=0$

Let us now consider two particular classes of solutions for Eq. (2.14). For one of these solutions, the right hand side of Eq. (2.14) vanishes for any value of the scale factor. This corresponds to the solution (2.21) for $\Delta =0$, $\sigma _0=0$ and ${\lambda _{\mathrm {c}}=0}$. Consequently, we have

3.5 $$\begin{aligned} \lambda _{\mathrm {H}} = \frac{2}{\beta }(2\beta -1)^2~, \quad \lambda _{\mathrm {m}} = 8\beta -1~, \quad \lambda _\mathrm {c} = 0. \quad \quad \end{aligned}$$

Then, the corresponding solution for *E*(*x*) in this case can be written as

3.6 $$\begin{aligned} E^{2}(x)\ =\ Ax+B, \end{aligned}$$

where *A* and *B* are constants.

By applying the condition (2.6), and using Eq. (2.7), we obtain

3.7 $$\begin{aligned} B=1,\quad \quad A = 2\left( \frac{\Omega _{\mathrm {H}_0}}{\beta }-2\right) = -2(1+q_0) . \end{aligned}$$

Since we expect $q_0>-1$ (this can be proven from a rough estimation based on the $\Lambda$CDM model), from the right hand side of Eq. (3.7), we expect *A* to be always negative. Positiveness of $E^2$ in Eq. (3.6) implies that, *x* always lies in the range $-\infty <x\le \frac{B}{|A|}$.

By using Eq. (3.6) in the relation $dx/dt=H=H_{0}E$ and integrating both sides, we obtain, for negative values of *A*, a relation for the time dependence of *x*, as

3.8 $$\begin{aligned} x(t)=-\frac{|A|H_{0}^{2}}{4}\left( t-t_{0}-\frac{2}{|A|H_{0}}\right) ^{2}+ \frac{1}{|A|}\ , \end{aligned}$$

where $t_{0}$ denotes the present time for which $x(t_{0})=0$. Notice that, we have set $B=1$ in the above relation. By taking the time derivative of *x* , and replacing it on the left hand side of equation $dx/dt=H_{0}E$, we obtain the dimensionless Hubble parameter

3.9 $$\begin{aligned} E(t)\ =\frac{A}{2}H_{0}(t-t_{0})+1\ . \end{aligned}$$

Consequently, the time derivative of the Hubble rate (3.9) reads

3.10 $$\begin{aligned} \dot{E}(t)\ =\frac{A}{2}H_{0}, \end{aligned}$$

which is constant during the evolution of the universe.

Since $-\infty <x\le \frac{1}{|A|}$ in this case, at $t_{b}$ where

$$\begin{aligned} t_{b}\ =\ t_{0}+\frac{2}{|A|H_{0}}~, \end{aligned}$$

*x*(*t*) reaches its upper limit $x=\frac{1}{|A|}$, where the Hubble rate vanishes, and the first time derivative of the Hubble rate, Eq. (3.10), remains constant. Thus, at this point the universe hits a bounce. Hereafter, the universe starts to collapse during an *infinite* time, and as $x\rightarrow -\infty$, for which $a\rightarrow 0$, the Hubble rate (3.9) diverges while its time derivative (3.10) remains finite. This represents a new abrupt event for the future fate of the universe which happens at $a=0$, and is different from the big bang singularity (for which the Hubble rate and its time derivative diverge). This abrupt event is smoother than a big bang: we named it the “*little sibling of the big bang*” (LSBB).

By using Eq. (2.1) and the total conservation equation $\dot{\rho }+3H(1+\omega _{\mathrm {tot}})\rho =0$, the total EoS $\omega _{\mathrm {tot}}=p_{\mathrm {H}}/(\rho _{\mathrm {H}}+\rho _{\mathrm {m}})$ reads

3.11 $$\begin{aligned} \omega _\mathrm{tot}= & {} \ -1-\frac{1}{3E^{2}}\frac{dE^{2}}{dx} \nonumber \\= & {} \ -1-\frac{A}{3(Ax+1)}\ \cdot \end{aligned}$$

In the limit $x\rightarrow -\infty$, the total EoS $\omega _\mathrm{tot}$ tends to $-1$. We would like to stress that the evolution of the universe is symmetric with regards to the bounce. Therefore, the universe evolves from a LSBB to a bounce and recollapse heading back to a LSBB.

The second class of solution is the case in which parameters $\lambda _{\mathrm {H}}$ and $\lambda _{ \mathrm {m}}$ satisfy the conditions (3.5) but $\lambda _{\mathrm {c}}\ne 0$. In this case Eq. (2.14) reduces to

3.12 $$\begin{aligned} \frac{d^{2}E^{2}}{dx^{2}}\ =\ -\frac{2}{\beta }\lambda _{\mathrm {c}}\ . \end{aligned}$$

With respect to the cosmic time *t*, Eq. (3.12) becomes

3.13 $$\begin{aligned} \ddot{E}(t)-\omega ^2 E(t)=0\ , \end{aligned}$$

where, $\omega ^2=-\frac{\lambda _{\mathrm {c}}}{\beta }H_{0}^2$. By imposing the conditions (2.6) and (2.7), the dimensionless Hubble rate reads

3.14 $$\begin{aligned} E(t)\ =\ C_{+}\exp (\omega t)+C_{-}\exp (-\omega t)\ , \end{aligned}$$

where, from Eq. (2.6) at the present time $t=t_0$, we get

3.15 $$\begin{aligned} C_\pm= & {} \ \frac{1}{2}\Big [1 \mp \frac{H_0}{\omega }(1+q_0)\Big ]\exp (\mp \omega t_0)\ . \end{aligned}$$

Moreover, the scale factor is given by^2^

3.16 $$\begin{aligned} a(t) = a_{0} \exp \left[ \frac{H_{0}}{\omega }C_{+}e^{\omega t}-\frac{H_{0}}{\omega }C_{-}e^{-\omega t}\right] . \quad \quad \end{aligned}$$

Here, $a_{0}$ is a constant of integration. Notice that, as $t\rightarrow +\infty$ the energy density (2.2) of the universe diverges.

Likewise, the Hubble rate blows up in this case.

In order to better understand the behaviour of the Hubble rate (3.12), it is convenient to write its solution in the following form:

3.17 $$\begin{aligned} E^{2}(x)\ =\ -\frac{\lambda _{\mathrm {c}}}{\beta }\left( x-x_{1}\right) \left( x-x_{2}\right) , \end{aligned}$$

where $x_{1}$ and $x_{2}$ are defined as

3.18 $$\begin{aligned} x_{1}&:= -\frac{\beta }{\lambda _{\mathrm {c}}}\left( (1+q_{0})+\sqrt{ (1+q_{0})^{2} +\frac{\lambda _{\mathrm {c}}}{\beta }}\right) , \end{aligned}$$

3.19 $$\begin{aligned} x_{2}&:= -\frac{\beta }{\lambda _{\mathrm {c}}}\left( (1+q_{0})-\sqrt{ (1+q_{0})^{2} +\frac{\lambda _{\mathrm {c}}}{\beta }}\right) . \end{aligned}$$

For $\lambda _{\mathrm {c}}<0$, the only physically interesting situations takes place when *x* belongs to the range $-\infty<x<x_{2}$: this describes a universe that starts its evolution at an infinite time in the past (where $x\rightarrow -\infty$ and $a\rightarrow 0$), at which the Hubble rate, *E*(*x*), and its time derivative, $\dot{E}$, diverge. Therefore, the universe hits a new abrupt event in this case which is similar to the big bang singularity, but which occurs at an infinite cosmic time. We thus name this new abrupt event as the “*little bang*” (LB).After this point, the universe expands till $x=x_2$ and then it bounces back to a LB. The other situation, $x_1\le x$, corresponds to an expansion that starts at a bounce when $x= x_1$ and heads to a little rip, when $x\rightarrow +\infty$ and $t\rightarrow +\infty$ [43–50]. Finally, when $\lambda _\mathrm{c}>0$, there is a unique Lorentzian solution that interpolate between two bounces located at $x=x_1=x_2$.

###  Interacting HRDE model

In this section, and in order to illustrate our purpose, we consider an interacting HRDE model with only the arbitrary parameters $\lambda _{\mathrm {H}}$ and $\lambda _{\mathrm {m}}$. By introducing a new quantity *r*, the ratio between the energy densities of CDM and HRDE [41, 42]:

3.20 $$\begin{aligned} r := \frac{\rho _{\mathrm {m}}}{\rho _{\mathrm {H}}}\ =\ \frac{\Omega _{ \mathrm {m}}}{\Omega _{\mathrm {H}}}\ , \end{aligned}$$

and using the conservation Eqs. (2.9) and (2.10), we get

3.21 $$\begin{aligned} \frac{dr}{dx} = (r+1)\frac{Q}{H\rho _{\mathrm {H}}}+3r\omega _{\mathrm {H}}\ . \end{aligned}$$

By substituting the interaction $Q=H(\lambda _{\mathrm {m} }r+\lambda _{\mathrm {H}})\rho _{\mathrm {H}}$ in Eq. (3.21), we obtain

3.22 $$\begin{aligned} \frac{dr}{dx} = (\lambda _{\mathrm {m}}+1)r^{2}+\left( \lambda _{\mathrm {H} }+\lambda _{\mathrm {m}}+1-\frac{2}{\beta }\right) r +\lambda _{\mathrm {H}}\cdot \nonumber \\ \end{aligned}$$

The solutions for the differential equation (3.22) depend on the sign of the discriminant $\Delta$, Eq. (2.20), which can be rewritten as

3.23 $$\begin{aligned} \Delta \ :=\ \alpha \big (\beta -\beta _{1}\big )\big (\beta -\beta _{2}\big ), \end{aligned}$$

where $\alpha$, $\beta _{1}$ and $\beta _{2}$ are:

3.24 $$\begin{aligned}&\alpha = (\lambda _{\mathrm {H}}+\lambda _{\mathrm {m}}+1)^{2}-4\lambda _{ \mathrm {H}}(\lambda _{\mathrm {m}}+1) ~, \end{aligned}$$

3.25 $$\begin{aligned}&\beta _{1} = \frac{2}{\alpha }\left[ \lambda _{\mathrm {H}}+\lambda _{ \mathrm {m}}+1 + 2\sqrt{\lambda _{\mathrm {H}}(\lambda _{\mathrm {m}}+1)}\right] , \end{aligned}$$

3.26 $$\begin{aligned}&\beta _{2} = \frac{2}{\alpha }\left[ \lambda _{\mathrm {H}}+\lambda _{ \mathrm {m}}+1 - 2\sqrt{\lambda _{\mathrm {H}}(\lambda _{\mathrm {m}}+1) }\right] . \end{aligned}$$

To get the physical solutions of Eq. (3.22), it is helpful to distinguish three cases $\Delta >0, \Delta =0$ and $\Delta <0$:

1. When $\Delta >0$, the parameter $\beta$ fulfils $\beta >\beta _{1}$ or $\beta <\beta _{2}$ (where $\beta _{2}<\beta _{1}$). In this case, the solution of the differential equation (3.22) reads,
  3.27 $$\begin{aligned} \quad \quad r(x) = -\frac{\mathbf {b}}{2\mathbf {a}} + \frac{\sqrt{\Delta }}{2\mathbf {a}{\beta }} \tanh \Big (-\frac{\sqrt{\Delta }}{2{\beta }}(x+k_1)\Big ), \end{aligned}$$
  where $\mathbf {a} := \lambda _{\mathrm {m}}+1$, $\mathbf {b}:=\lambda _{\mathrm {m }}+\lambda _{\mathrm {H}}+1-\frac{2}{\beta }$ and $k_1$ is a constant of integration. As $x\rightarrow \infty$, the ratio *r*(*x*) in Eq. (3.27) tends to a constant:
  3.28 $$\begin{aligned} \quad \quad r(x\rightarrow \infty ) = -\frac{\sqrt{\Delta }+{\beta }(\lambda _{\mathrm {H} }+\lambda _{\mathrm {m}} +1-\frac{2}{\beta })}{2{\beta }(\lambda _{\mathrm {m}}+1)} \cdot \nonumber \\ \end{aligned}$$
  A physical solution implies that *r*(*x*) must be always a positive valued function, that is, $r(x\rightarrow \infty )\ge 0$; this corresponds to ${\beta }(\lambda _{\mathrm {H} }+\lambda _{\mathrm {m}}+1-\frac{2}{\beta })+\sqrt{\Delta }\le 0$. Consequently, this relation imposes a constraint on the holographic parameter $\beta < \frac{2}{1+\lambda _{\mathrm {H}}+\lambda _{\mathrm {m}}}$. Since $\beta _{1}$ is always larger than $\frac{2}{1+\lambda _{\mathrm {H}}+\lambda _{\mathrm {m}}}$, the positivity of the function $\Delta$ implies that $\beta$ must be on the range
  3.29 $$\begin{aligned} \beta<\beta _{2}<\frac{2}{1+\lambda _{\mathrm {H}}+\lambda _{\mathrm {m}}} <\beta _1 \ , \end{aligned}$$
  for the solution (3.27) to be meaningful. In the far future (as $x\rightarrow +\infty$), by setting $\lambda _{\mathrm {H}}=0$ and for the range of the parameters satisfying $0<\beta <\frac{2}{1+\lambda _{\mathrm {m}} }$, the ratio of the energy densities *r*(*x*) vanishes ($r\rightarrow 0$) and the budget content of the universe becomes dominated by the HRDE, as would be expected. On the other hand, if $\beta \ge \frac{2}{1+\lambda _{\mathrm {m}} }$, the ratio *r*(*x*) becomes negative which is not physically possible. While by setting $\lambda _{\mathrm {m}}=0$, in the far future, *r*(*x*) approaches the value
  3.30 $$\begin{aligned} \quad \ r(x\rightarrow +\infty )\ =\ -\frac{1}{2}\Big (\lambda _{\mathrm {H}}-\frac{2}{\beta }+1+\frac{\sqrt{\Delta } }{\beta }\Big ). \quad \end{aligned}$$
  Since *r*(*x*) is always positive, the parameter $\beta$ is constrained to fulfil $\beta <\frac{2}{1+\lambda _{\mathrm {H}}}$.
2. For the case $\Delta =0$, the possible values of $\beta$ are $\beta =\beta _{1}$ or $\beta =\beta _{2}$. In this case, the solution of Eq. (3.22) for *r*(*x*) is given by
  3.31 $$\begin{aligned} r(x)&=\ -\frac{b}{2\mathbf {a}} - \frac{1}{\mathbf {a}\left( x+k_2\right) }\ , \end{aligned}$$
  where $k_2$ is a constant of integration. On the limit $x\rightarrow +\infty$, the solution (3.31) tends to
  3.32 $$\begin{aligned} r(x\rightarrow \infty )\ =\ -\frac{(\lambda _{\mathrm {H}}+\lambda _{\mathrm {m}}+1-\frac{2}{ \beta })}{2(\lambda _{\mathrm {m}}+1)}\ \cdot \end{aligned}$$
  Again *r*(*x*) must be positive, then, the holographic parameter must be on the range $\beta <\frac{2}{1+\lambda _{\mathrm {H}}+\lambda _{\mathrm {m}}}$. Since $\beta _{1}$ is larger than $\frac{2}{1+\lambda _{\mathrm {H}}+\lambda _{ \mathrm {m}}}$, the only possible range for the holographic parameter in this case is
  3.33 $$\begin{aligned} \beta =\beta _{2}<\frac{2}{1+\lambda _{\mathrm {H}}+\lambda _{\mathrm {m}}} <\beta _1. \end{aligned}$$
3. Finally, if $\Delta <0$ then $\beta _{2}<\beta <\beta _{1}$. The solution of Eq. (3.22) in this case is given as
  3.34 $$\begin{aligned} \quad \quad r(x) = -\frac{b}{2\mathbf {a}}+\frac{\sqrt{\left| \Delta \right| }}{2\mathbf {a}{\beta }} \tan \Big ( \frac{\sqrt{\left| \Delta \right| }}{2{\beta }}(x+k_3)\Big ) , \end{aligned}$$
  where $k_3$ is an integration constant. Equation (3.34) shows that when the argument of the ‘tangent’ term reaches the values $\pm \frac{\pi }{2}+n\pi$ where $n\in \mathbb {Z}$, the ratio *r* diverges with positive and negative signs, respectively. The former corresponds to a universe filled with CDM at late time, which does not match with the cosmological observations. In addition the latter limiting case corresponds to a negative ratio *r*(*x*) for the energy densities of the universe. Therefore, the solution given by Eq. (3.34) (that is, the solution provided by the case $\Delta <0$) is not physically relevant.

We will henceforth, study the late time behaviour of the universe predicted by the two physical solutions corresponding to $\Delta >0$ and $\Delta =0$.

#### The case $\Delta >0$

The dimensionless Hubble rate *E*(*x*) can be obtained by using Eq. (2.17) in which $\Lambda _\mathrm{c}=0$ and $\sigma _{\pm }$ are given by

3.35 $$\begin{aligned} \sigma _\pm \ =\ \frac{2+(\lambda _{\mathrm {m}}-\lambda _{\mathrm {H} }-7)\beta \pm \beta \sqrt{\Delta }}{2\beta }\ , \end{aligned}$$

with $\Delta \ne 0$ given by Eq. (3.23). Moreover, $\mathbf {A}_{\pm }$ reads

3.36 $$\begin{aligned} \mathbf {A}_{\pm }\ =\ \pm \frac{(3-4q_{0}+\lambda _{\mathrm {H}}-\lambda _{\mathrm {m} }\pm \sqrt{\Delta })\beta -2}{2\beta \sqrt{\Delta }}\ . \end{aligned}$$

When $\Delta >0$, the Hubble rate (2.17) at late time becomes

3.37 $$\begin{aligned} E^{2}(x)=\mathbf {A}_{+}\exp \Big [\frac{2+(\lambda _{\mathrm {m}}-\lambda _{\mathrm {H} }-7)\beta +\beta \sqrt{\Delta }}{2\beta }x\Big ] . \quad \quad \quad \end{aligned}$$

For the two ranges of the parameter $\beta$ in which $\beta <\frac{2}{ 7+\lambda _{\mathrm {H}}-\lambda _{\mathrm {m}}}$ or $\frac{2}{7+\lambda _{ \mathrm {H}}-\lambda _{\mathrm {m}}}<\beta <\frac{3-\lambda _{\mathrm {m}}}{ 6+2(\lambda _{\mathrm {H}}-\lambda _{\mathrm {m}})}$, the argument of the exponential term in Eq. (3.37) is positive. In this case, the universe will undergo a BR singularity in the far future. By integrating Eq. (3.37), we can find the behaviour of the scale factor *a*(*t*) at late times as

3.38 $$\begin{aligned} a(t)\ =\ a_0\left[ \frac{2}{2-\sigma _+H_0\sqrt{\mathbf {A}_+}(t-t_0)}\right] ^{\frac{2}{ \sigma _+}}\ . \end{aligned}$$

This indicates that the BR occurs at $t_{\mathrm {BR}}$ where

3.39 $$\begin{aligned} t_{\mathrm {BR}}\ =\ t_{0}+\frac{\left( 4\beta /H_{0}\sqrt{\mathbf {A}_{+}}\right) }{ 2+(\lambda _{\mathrm {m}}-\lambda _{\mathrm {H}}-7)\beta +\beta \sqrt{\Delta }}\ , \end{aligned}$$

and the scale factor diverges as $a(t_{\mathrm {BR}})\rightarrow +\infty$. On the other hand, for the range of the holographic parameter such that $\beta > \frac{3-\lambda _{\mathrm {m}}}{6+2(\lambda _{\mathrm {H}}-\lambda _{\mathrm {m} })}$, the argument of the exponential term is negative, the Hubble rate vanishes and the universe converges to a Minkowski state in the far future. In addition, for $\beta =\frac{3-\lambda _{\mathrm {m}}}{6+2(\lambda _{ \mathrm {H}}-\lambda _{\mathrm {m}})}$ and $\beta \ne \frac{2}{7+\lambda _{ \mathrm {H}}-\lambda _{\mathrm {m}}}$ the Hubble rate (3.37) reduces to a constant $E^{2}=\mathbf {A}_{+}=const.$, indicating that the universe approaches a de Sitter state at late time.

By setting $\lambda _{\mathrm {H}}=0$, Eq. (2.17) reduces to

3.40 $$\begin{aligned} E^{2}(x)= & {} \Big \{\beta \Big (\frac{\lambda _{\mathrm {m}} +2q_{0}-1}{\beta (1+\lambda _{\mathrm {m}} )-2}\Big ) \exp \Big [\Big (\frac{2}{\beta }-(\lambda _{\mathrm {m}}+1)\Big )x\Big ] \nonumber \\&+ \, 2\Big (\frac{\beta (1-q_{0})-1}{\beta (1+\lambda _{\mathrm {m}})-2}\Big )\Big \} \exp \big [(\lambda _{\mathrm {m}}-3)x\big ]. \end{aligned}$$

Now, we summarise the properties of the solution (3.40), for the case $0<\beta <\frac{2}{1+\lambda _{\mathrm {m}}}$:

1. For the case $\lambda _{\mathrm {m}}\ge 3$, the holographic parameter $\beta$ is within the range $\beta <\frac{2}{1+\lambda _{\mathrm {m}}}\le \frac{1}{2}$ . In this range, the first term in Eq. (3.40) becomes negative; $\frac{\lambda _{\mathrm {m}}+2q_{0}-1}{\beta (1+\lambda _{\mathrm {m}})-2}<0$, and the second one becomes positive; $\frac{ \beta (1-q_{0})-1}{\beta (1+\lambda _{\mathrm {m}})-2}>0$. Thus, the Hubble rate decreases and vanishes at $x_b$ which means that the universe hits a bounce at
  3.41 $$\begin{aligned} x_b=\frac{\beta }{2-\beta (1+\lambda _{\mathrm {m}})}\ln \left[ \frac{2\big (\beta (q_{0}-1)+1\big ) }{\beta (\lambda _{\mathrm {m}}+2q_{0}-1)}\right] . \end{aligned}$$
  In the far future, after the bounce ($x\rightarrow -\infty$), the dimensionless Hubble rate vanishes and the universe tends to a Minkowski state.
2. For the case $\lambda _{\mathrm {m}} <3$, the holographic parameter $\beta$ satisfies the condition $\beta<\frac{1}{2}<\frac{2}{1+\lambda _{\mathrm {m}} }$ and we have always $\frac{\beta (1-q_{0})-1}{\beta (1+\lambda _{\mathrm {m}})-2}>0$. Two possibles late time behaviour of the universe can be found. The case $\lambda _{\mathrm {m}}<1-2q_0 <3$, i.e. $\frac{\lambda _{\mathrm {m}}+2q_{0}-1}{\beta (1+\lambda _{\mathrm {m}})-2}>0$, where the universe hits a BR singularity in the far future. The case $1-2q_0<\lambda _{\mathrm {m}}<3$, i.e. $\frac{ \lambda _{\mathrm {m}}+2q_{0}-1}{\beta (1+\lambda _{\mathrm {m}})-2}<0$, where the dimensionless Hubble rate decreases and vanishes at $x_b$ (given in Eq. (3.41)) and the universe bounces at this point. In the far future, after the bounce ($x\rightarrow -\infty$), the Hubble rate vanishes and the universe tends to a Minkowski state.

The late time behaviour of the Hubble rate *E*(*x*) for the case $\lambda _{\mathrm {m}}=0$, is the same as the general one given by Eq. (3.37); see Table 1 for more details.

#### The case $\Delta =0$

In this case, where $\beta =\beta _{1}$ or $\beta =\beta _{2}$, the Hubble rate is given by Eq. (2.21):

3.42 $$\begin{aligned} E^{2}\ =\ (\mathbf {A}_{0}+\mathbf {A}_{1}x)\exp \left( \frac{2+(\lambda _{\mathrm {m}} -\lambda _{\mathrm {H}}-7)\beta }{2\beta }x\right) , \end{aligned}$$

where $\mathbf {A}_{0}=1$ and $\mathbf {A}_{1}$ are defined in Eq. (2.24) as $\mathbf {A}_1=-\sigma _0-2(1+q_0)$, where $\sigma _0$ is the argument of the exponential term in the equation above. The total EoS of the universe in this case reads

3.43 $$\begin{aligned} \omega _\mathrm{tot}\ =\ -1-\frac{\mathbf {A}_{1}}{3(1+\mathbf {A}_{1}x)}+\frac{\sigma _0}{3} \ , \end{aligned}$$

which converges to $-1+\frac{\sigma _0}{3}$ as $x\rightarrow -\infty$.

Following the discussion in item 3.3 above, we consider only the case $\beta =\beta _{2}$. For $\beta <\frac{2}{7+\lambda _{\mathrm {H}}-\lambda _{\mathrm {m}}}$, the argument of the exponential term is always positive ($\sigma _0>0$), while $\mathbf {A}_{1}<0$. This indicates that the universe undergoes a bounce at some $x_b=\frac{1}{|\mathbf {A}_1|}$ in the future. Hereafter, the universe starts to collapse and as $x\rightarrow -\infty$ it converges into a Minkowski state.

For $\frac{2}{7+\lambda _{\mathrm {H}}-\lambda _{\mathrm {m}}}<\beta < \frac{2}{1+\lambda _{\mathrm {H}}+\lambda _{\mathrm {m}}}$ (cf. Eq. (3.33)), the argument of the exponential term is always negative ($\sigma _0<0$):

if $|\sigma _0|>2(1+q_0)$, then $\mathbf {A}_{1}>0$; the exponential term decays faster than the linear term $\mathbf {A}_{1}x$, thus, the universe tends to a Minkowski state in the far future.

However, when $|\sigma _0|<2(1+q_0)$ (so that $\mathbf {A}_{1}<0$), the universe will bounce at $x_b=\frac{1}{|\mathbf {A}_1|}$ in the future, afterwards, it will start collapsing and in an infinite time, where $x\rightarrow -\infty$ (i.e., $a\rightarrow 0$), the Hubble rate and its time derivative diverge. Therefore, the universe meets a LB at late time. Notice that, in this case ($\sigma _0<0$), the matter content of the universe at late time behaves as phantom matter.

In the case of interaction parameter $\lambda _{\mathrm {m}}=0$, if $\lambda _{\mathrm {H}} >9$ and ${\sigma }_{0}>-2(1+q_{0})$, the term in the bracket in Eq. (3.42) is negative, while the argument of the exponential on the same equation is positive (for $x>0$). Therefore, the universe bounces in the future at some $x_b=1/|\mathbf {A}_{1}|$. After the bounce, the universe starts collapsing towards $x<0$. Hereafter, the term in the bracket will be always positive, while the argument of the exponential term will evolve negatively. Whence, in the far future ($t\rightarrow +\infty$), as $x\rightarrow -\infty$ the Hubble rate tends to zero and the universe tends to a flat Minkowskian state.

Finally, for the choice of $\lambda _{\mathrm {H}}=9$, we get ${\sigma }_{0}=0$, $\beta =1/8$ and $\mathbf {A}_1=-2(1+q_0)<0$. In this case, Eq. (3.42 ) reduces to Eq. (3.6), we have

$$\begin{aligned} E^{2}(x)\ =\ 4\left( 4\Omega _{\mathrm {H}_{0}}-1\right) x+1. \end{aligned}$$

This implies a bouncing scenario for the universe at $x=x_b=\frac{1}{ 2(1+q_0)}$. Hereafter, the universe starts to collapse during an infinite time, and as *x* tends to $-\infty$ the scale factor tends to zero, the Hubble rate diverges while its time derivative remains finite; $\dot{E}=2H_0(4\Omega _{\mathrm {H}_{0}}-1)$. Therefore, in the far future ($t\rightarrow +\infty$), the universe hits a LSBB abrupt event. In summary, what happens is that the universe evolves from a LSBB in the past, expands until it bounces and heads back to a LSBB.

Whenever $\mathbf {A}_{1}<0$, the dimensionless parameter $\Omega _{\mathrm {H}_0}$ satisfies $\Omega _{\mathrm {H}_0}<\frac{1}{4}$. Consequently, by substituting $\beta =1/8$ in the Eq. (2.16) we find that the EoS lies in the range

$$\begin{aligned} -\frac{16}{3}<\omega _{\mathrm {H}}<-4~, \quad \quad (0<t<+\infty ). \nonumber \end{aligned}$$

This relation implies that, the universe starts from a LSBB in the past with EoS for the HRDE $\omega _{\mathrm {H}}=-4$, and reaches a bounce when $\omega _{\mathrm {H}}=-\frac{16}{3}$ at some $t_b$ in the future. After the bounce, the universe will collapse and will hit a LSBB abrupt event in the far future ($t\rightarrow +\infty$) with the same EoS, $\omega _{\mathrm {H}}=-4$, of the initial state. Since the EoS of the dark energy evolves in the region $\omega _{\mathrm {H}}<-1$, the corresponding interacting HRDE model represents a phantom-like behaviour, although its EoS is too negative to describe nowadays universe. Notice that, when the universe hits a LSBB in the far future, the total EoS, $\omega _{\mathrm {tot}}$, of the universe tends to $-1$ which has the same value for the total EoS at the initial LSBB abrupt event.

**Table 1:** Summary of the behaviours of the universe at late times, for the physical range of holographic parameters $\beta <\frac{1}{2}$, for different DM and DE interactions

Sec.	Interacting model	$\Delta$	$\beta$	$\lambda$	$\sigma _0, \sigma _{\pm }$	Late time behaviour
3.1	$\lambda _\mathrm{m}=\lambda _\mathrm{H}=0$	$\Delta >0$	$\beta =\frac{1}{2}$	–	$\sigma _-=-3$, $\sigma _+=0$	de Sitter
		$\Delta >0$	$\beta <\frac{1}{2}$	–	$\sigma _-=-3$, $\sigma _+>0$	BR
3.2	$\lambda _\mathrm{m}=8\beta -1$,	$\Delta =0$	$\beta <\frac{1}{2}$	${\lambda _\mathrm{c}=0}$	$\sigma _0=0$	LSBB
	$\lambda _\mathrm{H}=\frac{2}{\beta }(2\beta -1)^2$			$\lambda _\mathrm{c}\ne 0$		LR
3.3	$\lambda _\mathrm{m}\ne 0$, $\lambda _\mathrm{H}=0$	$\Delta =0$	$\beta <\frac{1}{2}$	–	$\sigma _0>0$	Minkowski
		$\Delta >0$	$\beta <\frac{2}{1+\lambda _\mathrm{m}}\le \frac{1}{2}$	$\lambda _\mathrm{m}\ge 3$	$\sigma _\pm >0$,	Minkowski
		$\Delta >0$	$\beta<\frac{1}{2}<\frac{2}{1+\lambda _\mathrm{m}}$	$\lambda _\mathrm{m}<1-2q_0<3$	$\sigma _{-}<0$, $\sigma _+>0$	BR
		$\Delta >0$	$\beta<\frac{1}{2}<\frac{2}{1+\lambda _\mathrm{m}}$	$1-2q_0<\lambda _\mathrm{m}<3$	$\sigma _{-}<0$, $\sigma _+>0$	Minkowski
3.3	$\lambda _\mathrm{m}=0$, $\lambda _\mathrm{H}\ne 0$	$\Delta =0$	$\beta <\frac{2}{1+\lambda _\mathrm{H}}$	$\lambda _\mathrm{H}<9$	${\sigma }_0<-2(1+q_0)<0$	Minkowski
		$\Delta =0$	$\beta <\frac{2}{1+\lambda _\mathrm{H}}$	$\lambda _\mathrm{H}<9$	$-2(1+q_0)<{\sigma }_0<0$	LB
		$\Delta =0$	$\beta <\frac{2}{1+\lambda _\mathrm{H}}$	$\lambda _\mathrm{H}>9$	$-2(1+q_0)<0<{\sigma }_0$	Minkowski
		$\Delta =0$	$\beta =\frac{1}{8}$	$\lambda _\mathrm{H}=9$	${\sigma }_0=0$	LSBB$^{\text {a}}$
		$\Delta >0$	$\beta <\frac{2}{7+\lambda _\mathrm{H}}$	$\lambda _\mathrm{H}>3$	${\sigma }_+>0$,	BR
					$${\sigma }_-<0$$	
		$\Delta >0$	$\frac{2}{7+\lambda _\mathrm{H}}<\beta <\frac{3}{2(3+\lambda _\mathrm{H})}$	$\lambda _\mathrm{H}>0$	${\sigma }_+>0$,	BR
					$${\sigma }_-<0$$	
		$\Delta >0$	$\frac{3}{2(3+\lambda _\mathrm{H})}<\beta <\frac{1}{2}$	all $\lambda _\mathrm{H}$	${\sigma }_\pm <0$	Minkowski
		$\Delta >0$	$\beta =\frac{3}{2(3+\lambda _\mathrm{H})}<\frac{1}{2}$	all $\lambda _\mathrm{H}$	${\sigma }_+=0$,	de Sitter
					$${\sigma }_-<0$$	
3.3	$\lambda _\mathrm{m}\ne 0$, $\lambda _\mathrm{H}\ne 0$	$\Delta =0$	$\beta =\beta _2<\frac{2}{7+\lambda _\mathrm{H}-\lambda _\mathrm{m}}$	–	$\sigma _0>0$	Minkowski
		$\Delta =0$	$\frac{2}{7+\lambda _\mathrm{H}-\lambda _\mathrm{m}}<\beta =\beta _2<\frac{2}{1+\lambda _\mathrm{m}+\lambda _\mathrm{H}}$	–	${\sigma }_0<-2(1+q_0)<0$	Minkowski
		$\Delta =0$	$\frac{2}{7+\lambda _\mathrm{H}-\lambda _\mathrm{m}}<\beta =\beta _2<\frac{2}{1+\lambda _\mathrm{m}+\lambda _\mathrm{H}}$	–	$-2(1+q_0)<{\sigma }_0<0$	LB
		$\Delta >0$	$\beta <\frac{2}{7+\lambda _\mathrm{H}-\lambda _\mathrm{m}}$ or	–	$\sigma _+>0$	BR
			$\frac{2}{7+\lambda _\mathrm{H}-\lambda _\mathrm{m}}<\beta <\frac{3-\lambda _\mathrm{m}}{6+2(\lambda _\mathrm{H}-\lambda _\mathrm{m})}$		$\sigma _-<0$	
		$\Delta >0$	$\beta >\frac{3-\lambda _\mathrm{m}}{6+2(\lambda _\mathrm{H}-\lambda _\mathrm{m})}$	–	${\sigma }_\pm <0$	Minkowski
		$\Delta >0$	$\beta =\frac{3-\lambda _\mathrm{m}}{6+2(\lambda _\mathrm{H}-\lambda _\mathrm{m})}$	–	$\sigma _+=0$, ${\sigma }_-<0$	de Sitter

$^{a}$ This is a specific case of the solution of Eq. (3.5) given in the Sect. 3.2 (cf. see the third row in the table above)

If $\beta =\frac{2}{7+\lambda _{\mathrm {H}}-\lambda _{\mathrm {m}}}$, for the particular case $\beta =\frac{2}{7}$, the interaction parameters are equal and the condition (3.5) is satisfied for $\lambda _{\mathrm {H}}=\lambda _{\mathrm {m}}=\frac{9}{7}$. Then, the Hubble rate is obtained from Eq. (3.6) as

3.44 $$\begin{aligned} E^{2}(x)\ =\ 2\left( \frac{7}{2}\Omega _{\mathrm {H}_{0}}-2\right) x+1. \end{aligned}$$

On the case where the holographic dimensionless parameter $\Omega _{\mathrm {H}_{0}}$ satisfies $\Omega _{\mathrm {H}_{0}}<4/7$, the universe undergoes a *bounce* followed by a LB in the far future. It can be shown that at the LB $\omega _{\mathrm {H}}(a\rightarrow -\infty )=-\frac{7}{4}$, whereas at the bounce it reads $\omega _{\mathrm {H}}(x=\frac{1}{|A_1|})=-\frac{7}{3}$. Therefore, $\omega _{\mathrm {H}}<-1$ and the corresponding interacting HRDE model has a phantom-like behaviour. Moreover, when the universe hits a LB in the far future, the EoS of HRDE tends to $-\frac{7}{4}$ again. In fact, there is a symmetric evolution with respect to the source; i.e. the universe heads from a LB to a bounce and then back to a LB.

All the solutions we have analysed in Sect. 3 are summarised in Table 1.

## Summary and conclusions

In this paper, we have considered a HRDE [14], as the dark energy component of the universe, coupled to the CDM component of the universe. As it is well known, in the absence of interaction ($Q=0$) and depending on the physically relevant range of the holographic parameter $\beta$, the late time universe will end up in a big rip (BR) singularity if $\beta <\frac{1}{2}$ [14]. We remind as well that those values of $\beta$ are consistent with the latest observations.

On this work, we are interested in investigating whether or not different interactions between HRDE and CDM components of the universe could resolve or smoothen the BR singularity. We considered different interaction functions such as $Q=\lambda _{\mathrm {m}} H\rho _{\mathrm {m}}$, $Q=\lambda _{\mathrm {H}} H\rho _{\mathrm {H}}$ and $Q= H(r\lambda _{\mathrm {m}}+\lambda _{\mathrm {H}})\rho _{\mathrm {H}}+{\lambda _{\mathrm {c}} H\rho _{\mathrm {c}}}$ to study the late time behaviour of the universe (*r* is defined in Eq. (3.20)).

In the presence of the general function $Q= H(r\lambda _{\mathrm {m}}+\lambda _{\mathrm {H}})\rho _{\mathrm {H}}+{\lambda _{\mathrm {c}} H\rho _{\mathrm {c}}}$, the corresponding differential equation (2.14) governing the dynamical evolution of the universe, possesses two types of generic solutions (cf. Eqs. (2.17) and (2.21)) depending on the discriminant function $\Delta$, given by Eq. (2.20).

For different choices of the interaction constants $\lambda _\mathrm{m}$, $\lambda _\mathrm{H}$, $\lambda _\mathrm{c}=0$, and the HRDE parameter $\beta$, the physically relevant solutions corresponding to $\Delta >0$ (cf. table 1) lead to an asymptotic behaviour of the universe with: (i) a BR singularity, (ii) a Minkowski or (iii) a de Sitter state in the far future (see table 1 for a summary of the late time behaviour of the different solutions).

In particular, we have shown that for proper combinations of $\lambda _\mathrm{H}$, $\lambda _\mathrm{m}$ and $\beta$, the BR singularity can be removed.

On the other hand, when $\Delta =0$ (and $\lambda _\mathrm{c}=0$), the general Hubble rate is given by Eq. (2.21). When $\sigma _0<0$ and $\mathbf {A}_1>0$ (or $\sigma _0>0$ and $\mathbf {A}_1<0$), the universe approaches a Minkowski state, asymptotically (cf. Table 1).

However, the case with $\mathbf {A}_1<0$ where $\sigma _0\le 0$, represents two new abrupt events in the far future of the universe. We next summarise carefully these two cases:

(i) For $\sigma _0=0$, the Hubble rate reads $E^2(x) =1- |\mathbf {A}_1|x$, while $\dot{E}=const.$. This solution bounces at $x_b=\frac{1}{|\mathbf {A}_1|}$. After this point, the universe starts to collapse and as $x\rightarrow -\infty$, the Hubble rate diverges while its time derivative remains finite. This corresponds to a new abrupt event, which is smoother than the big bang singularity and happens in an infinite cosmic time. We have called it the “little sibling of the big bang” (LSBB).
(ii) For $\sigma _0<0$, the Hubble rate reads $E^2 = \ (1- |\mathbf {A}_1|x)e^{-|\sigma _0|x}$, therefore $\dot{E} = -\frac{H_0}{2}[|\mathbf {A}_1|+|\sigma _0|(1- |\mathbf {A}_1|x)]e^{-|\sigma _0|x}$. This solution indicates that, the universe bounces first at $x_b=\frac{1}{|\mathbf {A}_1|}$, then will collapse, and as $x\rightarrow -\infty$, the Hubble rate and its time derivative diverge at an infinite time. Therefore, in the far future ($t\rightarrow +\infty$), the universe tends to another type of abrupt event which we have called the “little bang” (LB) (cf. table 1).

Finally, a similar analysis for the case $\Delta =0$ and $\sigma _0=0$, for a non-vanishing interaction parameters $\lambda _\mathrm{c}$, represents a universe which starts its evolution from an infinite past with a LB, and afterwards it expands until it bounces at a finite time in the future, and then it recollapses again to a LB; or it can start from a bounce and heads to a little rip at an infinite cosmic time. For $\lambda _\mathrm{c}>0$, there is a unique Lorentzian solution that interpolate between two bounces.

These new types of abrupt events arise only when an interaction between HRDE and CDM is present. We have further shown that, under these conditions the HRDE may have a phantom like behaviour.

## Footnotes

1 It is worth mentioning that the fate of the BR in the HRDE within a quantum cosmological formalism was analysed in [33] and proven to be harmless once appropriate boundary conditions on the wave function of the universe are imposed.

2 We thank the referee for reminding us this class of solutions.
